# Pharmacological effects of the phytochemicals of *Anethum sowa* L. root extracts

**DOI:** 10.1186/s12906-016-1438-9

**Published:** 2016-11-14

**Authors:** Md Moshfekus Saleh-e-In, Nasim Sultana, Md Nur Hossain, Sayeema Hasan, Md Rabiul Islam

**Affiliations:** 1 Department of Chemistry, Jahangirnagar University, Savar, 1342 Dhaka Bangladesh; 2 Institute of National Analytical Research and Services, BCSIR Laboratories, Bangladesh Council of Scientific and Industrial Research, Dhaka, 1205, Bangladesh; 3 Industrial Microbiology Research Division, Institute of Food Science and Technology, Bangladesh Council of Scientific and Industrial Research, Dhaka, 1205, Bangladesh; 4 Department of Microbiology, Jagannath University, Dhaka, 1100, Bangladesh

**Keywords:** *Anethum sowa* L, Antioxidant, Cytotoxicity, Gram-positive bacteria, Gram-negative bacteria, Phytochemical screening

## Abstract

**Background:**

*Anethum sowa* L. is widely used as an important spice and traditional medicinal plants to treat various ailments. On the basis of scientific ethnobotanical information, this study was undertaken to evaluate the antioxidant, antimicrobial and cytotoxic activity of the crude extracts of *Anethum sowa *L. roots as well as to identify the classes of phytochemicals by chemical tests.

**Methods:**

The antioxidant potential of the extracts was ascertained with the stable organic free radical (2, 2-diphenyl-1-picryl-hydrazyl). The agar well diffusion method was used to determine the susceptibility of bacterial and fungal strains of the crude extracts. The minimum inhibitory concentration (MIC) and minimum bactericidal concentrations (MBC) were determined by the microdilution test. Cytotoxic activities were screened using brine shrimps (*Artemia salina*) lethality assay. Finally, phytochemicals were profiled using standard procedures.

**Results:**

A preliminary phytochemical screening of the different crude extracts by methanol, ethyl acetate and chloroform showed the presence of secondary metabolites such as flavonoids, alkaloids, saponin, cardiac glycosides and tannins while cyanogenetic glycosides were not detected. The methanol, ethyl acetate and chloroform extracts displayed high antioxidant activity (IC_50_ = 13.08 ± 0.03, 33.48 ± 0.16 and 36.42 ± 0.41 μg/mL, respectively) in the DPPH assay comparable to that of the standard ascorbic acid and BHT (IC_50_ = 3.74 ± 0.05 and 11.84 ± 0.29 μg/mL). The cytotoxic activity of the crude ethyl acetate and chloroform extracts possessed excellent activity (LC_50_ = 5.03 ± 0.08, 5.23 ± 0.11 and 17.22 ± 0.14 μg/mL, respectively) against brine shrimp larvae after 24 h of treatment and compared with standard vincristine sulphate (LC_50_ = 0.46 ± 0.05 μg/mL). The extracts also showed good antimicrobial activity against both Gram-positive and Gram-negative bacteria when compared with two standard antibiotics ciprofloxacin and tetracycline.

**Conclusion:**

These results showed that the *Anethum sowa* root extracts are the important source of the antioxidant, antimicrobial and cytotoxic agent. So, further research is necessary to isolate and characterize of different phytoconstituents for pharmaceutical drug lead molecules and also to verify its traditional uses.

## Background

Plant derived natural products are known to play an important role in both drug discovery and chemical biology. The medicinal properties of plants have been investigated due to their potent pharmacological activities, low toxicity and economic viability [1, 2]. There is an abundance of medicinal plants throughout the world, but only limited numbers have been investigated for its biological and pharmacological properties [3]. Plant phytochemicals have a significant role in the plants defense mechanism and also important for their unambiguous physiological action in the human body. Specially the secondary metabolites are becoming a part of the integrated health care system as supportive and alternative medicines because of their therapeutic property [4]. Therefore, it is essential to study the medicinal plants so that the discovery of active natural products ingredient can be identified for healing diseases. Later on, the identified active ingredients could be synthesized in the laboratory.

*Anethum sowa* L. (Bengali-Shulfa) belonging to the family *Apiaceae* (*Umbelliferae*), is a common aromatic and spice herb known as Shulfa in Bangladesh and found as a Dill in Europe, Asia-temperate, Africa and Asian-tropical countries. Indigenous people consume it as a spice for a flavouring agent in culinary preparation. The herb grows ordinarily 2–2.5 ft. in height with small feathery leaves, tapped and branched roots [5, 6]. The green herb, seeds and its roots are used as folkloric medicine e.g. aromatic, carminative especially useful in flatulence, colic and hiccups of infants and children [7]. The insecticidal, ovicidal and synergistic activity of dill-apiol and essential oil of the seed part are well known [8]. Moreover, it was reported that seed essential oils are the potential source of antioxidant and also have antimicrobial and antispasmodic properties [9]. Recently the phytochemical screening, anti-depressant and analgesic effects of aqueous extracts by *Anethum graveolens* L. has been reported [10]. Abbas et al. [11] also reported the lipid-lowering of hydroalcoholic extract of *Anethum graveolens* L.

Antioxidants protect cells against damage caused by molecules known as free radicals. Free radicals result from an imbalance between the generation of Reactive Oxygen Species (ROS) and the antioxidant protection conferred by enzymatic systems. The oxidation induced by ROS can result in cell membrane disintegration, membrane protein damage and DNA mutation. Over-production of ROS can result in progression of many diseases, such as cancer, diabetes, liver injury, arteriosclerosis, cardiovascular disease and lead to aging-related disorders [12]. The quest for natural antioxidants for dietary, cosmetic and pharmaceutical uses have become a major industrial and scientific research challenge over the last two decades. Plant secondary metabolites, mainly in the form of phenolic and nitrogen compounds as well as carotenoids and ascorbic acid have been reported to exhibit antioxidant and anticancer activities [13]. Synthetic antioxidants are commonly used in processed foods which have side effects and also reported carcinogenic [14]. Currently, there has been a growing interest of many research groups to identify plant-derived natural antioxidant compounds that are pharmacologically potent and have nutritional and therapeutic value.

In recent years, multiple drug resistance in human pathogenic microorganism has been developed due to indiscriminate use of commercial antimicrobial drugs commonly used in the treatment of various diseases. Plant biomolecules (phytochemicals) have been reported to be alternatives to antibiotic resistance of human pathogens because of their proven effectiveness and availability [15]. Therefore, the search for new antimicrobial substances from various medicinal plants through isolation and characterization of their constituents is warranted.

Bioactive compounds are almost always toxic in high doses. Pharmacology is simply toxicology at a lower dose and toxicology is simply pharmacology at a higher dose. Bioactive compounds are often toxic to shrimp larvae. Hence, in vivo lethality to shrimp larvae can be used as a rapid, simple, preliminary bioassay for testing plant extracts and organic compounds which in most cases correlates reasonably well with cytotoxic and anti-tumor properties [16]. Moreover, this test is also considered to be very useful in determining various biological activities such as phototoxic, pesticidal, trypanocidal, enzyme inhibition and ion regulation activities [17].

Despite some folklore use of this plant, there is no scientific report documented in the information on its phytochemical, antioxidant and antimicrobial as well as cytotoxic properties of this plant to the best of our knowledge. In this study, we sought to interrogate the antioxidant, anti-microbial and cytotoxic effect compared with the commercial standard as well as the screening of phytoconstituents of *Anethum sowa* L. root extracts.

## Methods

### Plant material

Whole plants were collected from Keranigonj, Dhaka of Bangladesh after harvesting. The plant was authenticated by Dr. Sardar Nasir Uddin, PSO, Bangladesh National Herbarium, Dhaka, Bangladesh. Voucher specimen with Accession Number-31,282 was placed at the Herbarium. Shade dried roots were powdered in a grinding machine to 100 mesh and stored in the airtight high-density polyethylene bag for solvent extraction.

### Chemicals and reagents

The chemicals n-hexane, chloroform, ethyl acetate and methanol (Merck Germany) were used for solvent extraction. The laboratory grade petroleum ether (bp. 40-60 °C) was collected from fuel petrol by fractional distillation. 1,1-diphenyl-2-picrylhydrazyl (DPPH) was purchased from Sigma-Aldrich, Munich, Germany. Butylated Hydroxytoluene (BHT) and ascorbic acid (BDH, England) were used as reference standard for free radical scavenging assay. Ciprofloxacin (5 μg/disc), tetracycline (30 μg/disc) (Oxoid, England) and fluconazole (100 μg/mL) (AFC Bangladesh) were used as the reference standard and positive control for antibacterial screening assay. Vincristine sulfate (Merck, Germany) was used as a reference cytotoxic agent in the brine shrimp lethality test.

### Preparation of the extracts

The powder material (1.5 kg) of *Anethum sowa* L. root was extracted successively with petroleum ether (b.p. 40–60 °C), chloroform, ethyl acetate and methanol at room temperature. Extractions were carried out three times in 3–4 days of each solvent with frequent shaking. Removal of solvent from powder materials was done by filtration through cheesecloth and Whatman filter paper No. 1. The filtrate was then further concentrated under reduced pressure by a rotary evaporator (Buchi, R-15v) at low temperature. The chloroform, ethyl acetate and methanol extract were triturated with pet.ether (3 × 50 mL) of each time for 2 h at room temperature to get the fat free extract. The pet.ether soluble extracts were combined with pet.ether extract. On the other hand, the certain amount of root powdered sample of *Anethum sowa* L. was carried out in hot extraction process by Soxhlet apparatus with hexane at room temperature for 72 h.

### Qualitative phytochemical group tests

The extracts were subjected to qualitative screening for the detection of phytochemical groups by established methods [18–20]. In each test 10 % (w/v) solution of the extract was taken unless otherwise mentioned in the individual test.

### Antioxidant activity (DPPH assay)

The free radical scavenging effect of *A. sowa* root extracts, ascorbic acid (ASA) and tert-butyl-1-hydroxytoluene (BHT) were assessed with the stable scavenger DPPH with slight modifications of the method described by Brand-Williams [21]. Briefly, the calculated amount (about 2 mg) of different extracts, ASA and BHT were dissolved in methanol to get a mother solution having a concentration 1000 μg/mL. ASA and BHT were used as positive control. DPPH solution (40 μg/mL) was prepared in methanol. The solution was prepared in the amber reagent bottle, kept in the light-proof box with ice. The degree of DPPH-purple decolorization to DPPH-yellow indicated the scavenging efficiency of the sample. Serial dilutions were made using the mother solution to get different concentrations (200, 100, 50, 25, 12.5, 6.25, 3.125 and 1.562 μL). 200 μL of a sample solution (extracts or control) at different concentration and 800 μL methanol (total 1000 μL) were mixed with 1000 μL of a DPPH solution. Incubation was performed for 25 min for reaction at room temperature (25 °C) in the dark place. The absorbance was measured at 517 nm against methanol as blank by UV-Visible spectrophotometer (UV-VIS 1650, Shimadzu Corporation, Japan). The lower absorbance of the reaction mixture indicated higher free radical scavenging activity. The percentage of inhibitions were calculated from the equation: % inhibition = (1- ABS_sample_/ABS_blank_) × 100. Where ABS_blank_ is the absorbance of the DPPH solution (control) and ABS_sample_ for the sample or standard absorbance. Then the percentage of scavenging of the extract was compared with positive control.

### IC_50_ value of the extract

The IC_50_ value data were transformed into a straight line by means of a trend line fit linear regression analysis by MS Excel version 7 Software for Windows. The IC_50_ values were calculated as the concentration of each sample required to give 50 % DPPH radical scavenging activity from the graph (linear regression curve) by Excel 2007 Office Software. The lower IC_50_ value indicates the higher radical scavenging effect. The experiment was performed triplicate and the results were expressed as mean ± SD with 95 % confidence interval in every case.

### Cytotoxic activity by Brine Shrimp lethality bioassay

In vitro Brine Shrimp lethality bioassay of the dried extracts were exploited to detect cytotoxicity by the Mayer’s method [17]. Brine Shrimp (*Artemia salina*) eggs were hatched in a brine solution (3.8 % NaCl in distilled water) within 48 h. The samples were prepared by dissolving in DMSO solution (not more than 50 μL in 5 mL solution) and it was applied in 5 mL brine solution to attain the concentrations of 0.0195 to 10.0 μg/mL. DMSO (50 μL) used as negative control. Standard vincristine sulfate (VcS) (0.078 μg/mL to 10.0 μg/mL) was used as positive control. Approximately 10 matured nauplii were taken to each vial. The number of surviving nauplii was counted after 24 h. The lethal concentrations (LC_50_) and the dose-response data were calculated from the linear regression graph by Excel 2007 Software.

## Antimicrobial activity of the plant extracts

### Microorganism

Gram-positive (*Bacillus subtilis*, *Staphylococcus aureus*, *Bacillus cereus*, *Enterococcus faecalis*) and Gram-negative bacteria (*Salmonella typhi*, *Escherichia coli 12079*, *Escherichia coli 2799*, *Pseudomonas aeruginosa*, *Salmonella enteritidis*, *Acetobacter aceti*) were used for antibacterial screening and *Aspergillus niger, Candida albicans* and *Trichoderma sp.* were used for fungal studies. All the stock cultures were collected from the Industrial Microbiology Division, Bangladesh Council of Scientific and Industrial Research, Dhaka, Bangladesh.

### Media preparation and maintenance of bacteria and fungi

All the bacterial and fungal strains were grown and maintained on Mueller Hinton agar (Himedia, India) media at 37 °C and pH (7.3 ± 0.2). The bacteria and fungi were subcultured overnight Mueller Hinton broth, which was further adjusted to obtain turbidity comparable to McFarland (0.5) standard when required [22].

### Antibacterial screening by diffusion technique

The antibacterial activity of the dried extracts was determined by diffusion and dilution method [23] against 4 g-positive, 6 g-negative bacteria and 3 fungi species. The test microbes were taken from the broth culture with an inoculating loop and transferred to the test tubes containing 5.0 mL sterile distilled water. The bacterial suspension turbidity adjusted to McFarland standard number 0.5, in Mueller Hinton Broth (Himedia, India). A cotton swab was then used to inoculate the test tube suspension onto the surface of the Mueller Hinton agar plates and the plates were allowed to dry. The agar was allowed to set and harden. Holes were made by using a sterile cork borer from each petri-plate to ensure proper distribution of holes (cups) in the periphery and one in the center. Agar plugs were removed. Different cork borers were used for different test organisms. Two standard discs (6 mm in diameter) ciprofloxacin and tetracycline were transferred onto the agar surface by using sterile forceps. Each hole was impregnated with 40 μL of a sample solution containing 800, 1200 and 2000 μg sample. This was done also for methanol (negative control) as a blank. These plates were kept for an hour at a low temperature, so that the test materials were diffused into the surrounding medium by this time. The plates were then incubated at 37 °C ± 1 °C for 24 h. The diameter of the inhibition zone against each microorganism by plant extracts was measured by digital calipers (Deko Corporation, China, accuracy 0.02 mm and resolution 0.01 mm) and compared the results with standard antibiotic ciprofloxacin (5 μg/disc) and tetracycline (30 μg/disc) as a positive control.

### MIC and MBC determination by dilution technique

Minimum Inhibitory Concentrations (MIC) and Minimum Bactericidal Concentrations (MBC) of the plant extracts were carried out by the macro-dilution method [24, 25]. The plant extracts were dissolved in 30 % dimethyl sulfoxide (DMSO) to obtain 10 % (w/v) solution. For MIC test of the selected bacteria, the extracts were first diluted in sterilized Mueller-Hinton broth in screw capped tubes containing broth medium in the concentration of 1000, 500, 250, 125, 62.5 and 31.25 μg/mL. Bacterial suspensions of the test organism were prepared in sterilized Mueller-Hinton broth. 1 mL of the dilution was added to each sterilized screw capped tube containing 1 mL of sample suitably diluted in the sterilized broth medium to give a final volume of 2 mL. A culture medium without sample (solvent DMSO) was used as a negative control. Ciprofloxacin (100–0.191 μg/mL.) was used as positive control. Tubes were incubated aerobically at 37 °C for 24 h and the growth was indicated by turbidity. The lowest concentration preventing visible growth (no turbidity) was identified as the MIC and expressed in μg/mL. The complete absence of growth was considered as the MBC [26]. To confirm the results of the MBC, 10 μL of the experimental suspensions (withdrawn from the tubes with no growth) were subcultured on TSA (Tryptone Soy Agar) plates. The Plates were incubated at 37 °C ± 1 °C for 24 h. It showed no bacterial growth, which was taken as the MBC. Values were recorded as μg/mL.

### Antifungal assay

The antifungal activity test was performed by a similar procedure as an antibacterial activity test. But the PDA growth medium was used instead of NA medium. Positive control (Flucanozole 100 μg/mL) and extracts containing petri dishs were incubated at 25 ± 2 °C for 72 h. The MICs and MBCs of the selected fungi were also done by the micro dilution method as described in the antibacterial activity test.

### Statistical analysis

Statistical analysis of all data was performed by means of the Microsoft Excel 7.0 version for Windows. All data are presented as mean value ± standard deviation (*n* = 3). The values were considered significantly different at (*P* < 0.05).

## Results and discussion

### Plant extract

Successive extraction of *Anethum sowa* L. (1.5 kg) root powder yielded (%) 0.60, 0.35, 0.72, 1.14 and 1.40 by petroleum ether, chloroform, ethyl acetate, hexane and methanol respectively (Table 1). The highest yield (20.94 g) was obtained from methanol crude extract.

**Table 1:** *A. sowa* root extracts yield

	Crude extracts				
	PE	CE	EtOAc	Hex. Hot	MeOH
Yields in grams (g.)	9.03	5.24	10.70	2.85	20.94
Yields in percentage (%)	0.60	0.35	0.72	1.14	1.40
Petroleum ether (PE), chloroform (CE), ethyl acetate (EtOAc), hexane hot extract (Hex. Hot), methanol (MeOH)					

### Phytochemical screening of extracts

Qualitative phytochemical tests of *Anethum sowa* L. were performed for the methanol (MeOH), ethyl acetate (EtOAc), chloroform (CE), pet-ether (PE cold) and hexane (Hex.hot) extracts of the root. The results of various chemical tests for the detection and identification of chemical constituents were summarized in Table 2. In the present study, flavonoids, glucosides, cardiac glycosides and anthraquinone glycosides were present in MeOH, EtOAc and CE extracts, but in addition, the saponins were present only in MeOH extract. Steroids were found in all the extract except MeOH extract. On the other hand, all the groups were absent in PE (cold) and Hex.(hot) extracts except steroids. However, alkaloids, carbohydrates and tannins were highly present in the MeOH extract whereas, EtOAc extract showed slightly positive test for alkaloids and absent in CE extract. The carbohydrates showed slightly positive in CE and EtOAc extract.

**Table 2:** Phytochemical constituents of different extracts (PE, Hex, CE, EtOAc and MeOH) of *Anethum sowa* L. root

Metabolites	Tests					
		PE (Cold)	Hex (Hot)	CE	EtOAc	MeOH
Saponins	a) Frothing	a) (−)	a) (−)	a) (−)	a) (−)	a) (+++)
	b) Emulsification	b) (−)	b) (−)	b) (−)	b) (−)	b) (+++)
Flavonoids	a) Alkaline reagent Test	a) (−)	a) (−)	a) (+++)	a) (+++)	a) (+++)
	b) Lead acetate Test	b) (−)	b) (−)	b) (++)	b) (++)	b) (++)
Alkaloids	a) Mayers’ reagents	a) (−)	a) (−)	a) (−)	a) (+)	a) (+++)
	b) Hager’s Test	b) (−)	b) (−)	b) (−)	b) (+)	b) (++)
	c) Wagner’s Test	c) (−)	c) (−)	c) (−)	c) (+)	c) (++)
Steroids	a) Salkowski test:	a) (+++)	a) (+++)	a) (+++)	a) (+++)	a) (−)
	b) Liebermann-Burchard’s test	b) (+++)	b) (+++)	b) (+++)	b) (+++)	b) (−)
Carbohydrates	a) Molisch’s test	a) (−)	a) (−)	a) (+)	a) (+)	a) (+++)
	b) Fehling test for reducing sugar	b) (−)	b) (−)	b) (−)	b) (−)	b) (+++)
Glucosides	a) General test	a) (−)	a) (−)	a) (++)	a) (++)	a) (+++)
	b) Test for glucosides	b) (−)	b) (−)	b) (+)	b) (+)	b) (+++)
Cardiac glycosides	a) Keller Killiani test	a) (−)	a) (−)	a) (+++)	a) (+++)	a) (+++)
	b) Baljet Test	b) (−)	b) (−)	b) (+++)	b) (+++)	b) (+++)
Anthraquinone glycosides	a) Borntrager’s Test					
	i) O-glycoside	i) (−)	i) (−)	i) (++)	i) (++)	i) (+++)
	ii) C-glycoside	ii) (−)	ii) (−)	ii) (−)	ii) (−)	ii) (−)
Tannins	a) Gelatin-salt block test:	a) (−)	a) (−)	a) (−)	a) (−)	a) (+++)
	b) Lead acetate test	b) (−)	b) (−)	b) (−)	b) (++)	b) (+++)
	c) Condensed or Phlobatanins	c) (+++)	c) (+++)	c) (++)	c) (++)	c) (++)
Cyanogenetic glycosides	a) Sodium picrate test	a) (−)	a) (−)	a) (−)	a) (−)	a) (−)

(−) = negative (absent), (+) = Positive (slightly present), (++) = Positive (moderately present), (+++) = Positive (Abundantly present)

The value of medicinal plants is due to phytochemical constituents or secondary metabolites that synthesized by the plant body during the plants normal metabolic processes and plants use them to protect themselves against different pathogenic attack [27]. The optimal effectiveness of a medicinal plant may not be due to the one main active constituent, but may be due to the combined action of different compounds originally in the plant [28]. Cardiac glycosides work by inhibiting the Na^+^/K^+^ pump. This causes an increase in the level of sodium ions in the myocytes, which then leads to a rise in the level of calcium ions. This inhibition increases the amount of Ca^2+^ ion available for contraction of the heart muscle, improves cardiac output and reduces distention of the heart [29]. Cardiac glycosides were found to be present in MeOH, EtOAc and CE extracts, it can be aided in treatment for congestive heart failure and cardiac arrhythmia. Alkaloids are nitrogen-containing naturally occurring compound, commonly found to have antimicrobial properties due to their ability to intercalate with the DNA of the microorganisms [30]. Flavonoids and tannins are phenolic compounds and plant phenolics are a major group of compounds that act as primary antioxidants of free radical scavengers [31]. Flavonoids are also found to be effective antimicrobial substances in vitro against a wide array of microorganisms by inhibiting the membrane bound enzymes. They have been reported to possess substantial anti-carcinogenic and anti-mutagenic activities due to their anti-oxidant and-inflammatory properties. They are also active in reducing high blood pressure [30]. On the other hand, tannins play a major role as antihaemorrhagic agent and have been shown to have immense significance as antihyper cholesterol, hypotensive and cardiac depressant properties [32]. Glycosides serve as defense mechanisms against predation by many microorganisms, insects and herbivores [33]. Moreover, glycosides, flavonoids, tannins and alkaloids have hypoglycemic activities [34]. Saponins are another type of bioactive chemical constituents which are involved in plant disease resistant because of their antimicrobial activity [35]. On the other hand, saponins have been shown to possess both beneficial (cholesterol-lowering) and deleterious (cytotoxic; permeabilization of the intestine) properties [36]. Although, some saponins have been shown to be highly toxic under experimental conditions and acute poisoning is relatively rare both in animals and man [37]. Hypocholesterolemic and antidiabetic properties of saponins are also reported [38]. Medicinal plants are the best sources for chemical ingredients. Based on preliminary phytochemical studies, the results revealed that Methanol, ethyl acetate and chloroform crude extracts of *A. sowa* root contained larger amounts of bioactive secondary metabolites that may be potential as chemotherapeutic drugs.

### Antioxidant activity on DPPH radical

The free radical scavenging activity of the root extracts of *Anethum sowa* L. were measured by DPPH assay and the summarized results are shown in Table 3. All the extract showed a significant DPPH scavenging activity in a concentration dependent manner. The lower the IC_50_ value is, the greater the free radical scavenging activity is. For each sample eight concentrations (1.562-200 μg/mL) were tested. Methanol extract exhibited considerably higher DPPH radical scavenging activity (96.18 %) with the IC_50_ value of 13.08 μg/mL than other extracts and the lowest (33.71 %) DPPH scavenging rate was found in the hexane (hot) extract with the IC_50_ value of 8.33 mg/mL. The free radical scavenging activities of the extracts decreased in the order of methanol > ethyl acetate > chloroform > pet.ether(cold)>hexane(hot). The IC_50_ values were also shown in Table 3. The ethyl acetate and chloroform extract showed the significant radical scavenging activity of 87.11 and 74.97 % with the IC_50_ values of 33.48 μg/mL and 36.42 μg/mL respectively. The IC_50_ value of the extract was found to be very fair compared to the IC_50_ value of the reference standard ASA (3.74 μg/mL) and BHT (11.84 μg/mL) with the highest inhibition of 99.05 and 93.02 % respectively. Moreover, the methanol extract showed the same radical scavenging activity like a standard at the same concentrations.

**Table 3:** Antioxidant activity of different extract and standard (Average ± SD)

		Average % of scavenging of different extract and standard						
Dose Conc. (μg/mL)	Log Conc. (μg/mL)	Hexane (Hot. Ext.)	Pet. Ether (Cold Ext.)	Chloroform	Ethyl acetate	Methanol	BHT	Ascorbic acid
200	2.30	33.71 ± 0.33	35.91 ± 0.65	74.97 ± 0.89	87.11 ± 0.61	96.18 ± 0.31	93.02 ± 0.50	99.05 ± 0.21
100	2	22.26 ± 0.97	22.09 ± 0.45	70.23 ± 0.53	79.35 ± 0.44	87.34 ± 0.27	91.98 ± 0.19	98.35 ± 0.17
50	1.69	10.29 ± 0.37	13.26 ± 0.33	60.72 ± 0.38	57.41 ± 0.27	79.71 ± 0.60	85.51 ± 0.76	90.71 ± 0.60
25	1.39	8.57 ± 0.31	11.60 ± 0.29	44.63 ± 0.19	32.27 ± 0.68	66.46 ± 0.34	69.54 ± 0.60	85.31 ± 0.60
12.5	1.09	5.14 ± 0.18	8.29 ± 0.20	23.80 ± 0.51	22.58 ± 0.44	47.49 ± 0.93	55.87 ± 0.82	79.79 ± 0.18
6.25	0.79	1.71 ± 0.06	1.65 ± 0.04	16.08 ± 0.57	14.82 ± 0.54	33.53 ± 0.34	29.69 ± 0.97	69.67 ± 0.68
3.125	0.49	0	0	10.72 ± 0.38	9.01 ± 0.75	20.87 ± 0.53	25.63 ± 0.51	37.26 ± 1.12
1.562	0.19	0	0	5.36 ± 0.19	1.93 ± 0.09	12.65 ± 0.26	9.05 ± 0.51	27.02 ± 1.19
IC_50_ value		8.33 ± 0.21 mg/mL	4.74 ± 0.22 mg/mL	36.42 ± 0.41 μg/mL	33.48 ± 0.16 μg/mL	13.08 ± 0.03 μg/mL	11.84 ± 0.29 μg/mL	3.74 ± 0.05 μg/mL

Each value is the mean ± SD of three determinations (*n* = 3) and *p* < 0.05

Antioxidant activity depends on their scavenging powers which are useful for the management of many disorders like atherosclerosis, angina pectoris, neurodegenerative diseases and cancer diseases. Many synthetic antioxidants such as butylated hydroxy anisole (BHA) and butylated hydroxyl toluene (BHT) are commercially available and widely used, but they may possess some side effects and toxic properties to human health [39]. So, attention has been directed towards the natural antioxidants from botanical sources, especially plants. However, a significant correlation is observed between the antioxidant activity of herbs and the phytochemical content.

Plant phenolic compounds or poliphenols are the natural antioxidants which can act as a free radical scavenger, metal chelators and singlet oxygen quenchers and as a multi-functional activity [40]. The anti-oxidative potential of flavonoid depends on their chemical structure. The hydroxyl groups of phenolic compounds allow them to exert direct anti-oxidative activity and were found to play a vital role in stabilizing lipid peroxidation. The most active antioxidant compound is catechol, which possesses two hydroxyl groups in the ortho position. The first chain carrying peroxyl radical was being trapped by H-atom transfer from the labile phenolic O–H and the second by reaction with the resultant phenoxyl radical [40] and produced stable quinines upon oxidation. This property renders these phenolic compounds as efficient antioxidants.

The presence of phenolic constituents like flavonoids and tannins in the phytochemical study may have contributed to the observed antioxidant activity of this plant parts. Therefore, phenolic compounds in *Anethum sowa* root extracts are good electron donors and could terminate the radical chain reaction by converting free radicals to more stable products. The high antioxidant activity shown by the methanolic extract of the root suggests that it is a potential therapeutic agent for the control of oxidative damage caused by reactive oxygen species.

### Brine shrimp cytotoxicity assay

The brine shrimp cytotoxicity of the plant extract represents a rapid, inexpensive and simple bioassay technique for cytotoxic, anti-tumor and anticancer activity. This test is also considered to be phototoxic, pesticidal, trypanocidal, antitumor, enzyme inhibition and ion regulation activities. The brine shrimp cytotoxicity bioassay of *Anethum sowa* L. root extracts and that of the positive control vincristine sulphate are summarized in Table 4. The extract showed lethality in a dose-dependent manner. All the extracts exhibited significant toxicity towards brine shrimps at 24 h. The LC_50_ value of the methanol extract was found the highest cytotoxicity at the LC_50_ value of 5.03 μg/mL with the 100 % mortality at 400 and 200 μg/mL dose levels followed by ethyl acetate (5.23 μg/mL), chloroform (17.22 μg/mL), pet ether (cold) (48.71 μg/mL) and hexane (Hot) (51.90 μg/mL) extracts. The activity of ethyl acetate extract was found to almost same as methanol extract. In comparison to standard positive control vincristine sulphate (LC_50_ = 0.46 μg/mL), the ethyl acetate and methanol extract were found promising. Moreover, chloroform extract found moderate activity. On the other hand, hexane (hot) and pet ether (cold) extract demonstrate the lower activity. Higher mortality was observed in the every extract at 400 μg/mL concentration dose levels.

**Table 4:** Cytotoxic activity of different extract and standard (Average ± SD)

Average % of Mortality of different extract and standard							
						Vincristine	Sulphate
Log Conc. (μg/mL)	Pet. Ether (Cold Ext.)	Hexane (Hot. Ext.)	Chloroform	Ethyl acetate	Methanol	Log Conc. (μg/mL)	Average % of mortality
2.602	80.60±1.04	80.60±1.04	100±0.00	100±0.00	100±0.00	1	100±.00
2.301	70.21±1.11	70.21±1.11	90.85±0.83	100±0.00	100±0.00	0.698	90.85±0.83
2	59.95±1.60	59.95±1.60	70.65±1.83	91.06±1.15	93.17±0.27	0.397	80.12±1.62
1.698	48.48±2.62	46.08±0.60	59.95±1.60	81.71±1.66	84.55±1.19	0.096	60.51±0.88
1.397	39.48±0.88	35.13±1.59	46.08±0.60	81.88±1.91	73.48±1.31	−0.204	50±0.00
1.096	29.34±1.83	26.94±1.80	38.27±1.82	59.95±1.60	64.85±1.59	−0.505	40.55±0.95
0.795	19.39±1.05	20.47±0.81	30.25±0.43	50±0.00	52.79±2.44	−0.806	30±0.00
0.494	9.69±0.52	9.69±0.52	28.61±1.36	38.05±2.17	40±0.00	−1.107	20±0.00
0.193	10±0.00	10±0.0	20.47±0.81	30±0.00	30±0.00	−1.408	10±0.00
−0.107	0	0	8.58±0.43	20±0.00	20±0.00	−1.709	10±0.00
−0.408	–	–	–	–	20±0.00	–	–
−0.709	–	–	–	–	10±0.00	–	–
LC_50_ value (μg/mL)	48.71±0.70	51.90±0.45	17.22±0.14	5.23±0.11	5.03±0.08		0.46±0.05

Each value is the mean± SD of three determinations (*n*=3) and *p*<0.05

Bioactive compounds are almost always toxic in high doses. In the brine shrimp lethality assay, the degree of lethality was directly proportional to the concentration of the crude extracts of the *A. sowa* root. The cytotoxic activity is recommended weak at the LC_50_ is 500–1000 μg/mL, moderate at 100–500 μg/mL and strong at 0–100 μg/mL [41]. However, according to Mayer [17], extracts derived from natural products which have LC_50_ ≤ 1.0 mg/mL are known to possess toxic effects. Conclusively, our results showed that all the extracts could be regarded as toxic to brine shrimp larvae cells based on the LC_50_ values in the current study. The *A. salina* embryos are highly vulnerable to toxins at early developmental stages [42]. A dose dependent relationship was observed where the percent of toxicity increased with increase in the concentration of the extracts. Bioassay-guided fractionation offers special advantages for identification of medicinal plant extracts. Most often, a desired biological response is not due to one component but rather due to a mixture of bioactive plant components [43]. Özçelik reported [44] the cytotoxic effect of plants is principally contributed by the presence of secondary metabolites like alkaloid, glycosides, steroid, tannin and flavonoids. Steroids, flavonoids and saponins which may also responsible for the prominent cytotoxic effect in brine shrimp lethality bioassay [45]. It has been established that the cytotoxic compounds generally exhibit significant activity in the brine shrimp lethality assay. Preliminary phytochemical screening of the extracts showed the presence of alkaloids, tannins, saponins and flavonoids type compounds in methanol, ethyl acetate and chloroform extract prominently. This assay can be recommended as a guide for the detection of antitumour and pesticidal compounds because of its simplicity and low cost. Further toxicity studies on individual cell line are also suggested to confirm the anticancer effect of the *Anethum sowa* L. root extracts.

### Antimicrobial activity of *Anethum sowa* L. root extracts

The antibacterial activity of *Anethum sowa* L. root extracts was tested in vitro by using diffusion and liquid dilution method. The extracts were tested against 10 pathogenic bacteria. The antibacterial activity of the extracts was assessed by determining their ZI, MIC and MBC values and shown in Tables 5, 6, 7 and 8. The results revealed that all the Gram-positive organisms were resistant to the PE (Cold), Hex (Hot), CE and MeOH extracts at 400 μg/well concentration except *B. cereus* in CE extract. It was observed that EtOAc extract produced the highest zone of inhibition against the Gram-positive bacteria (12–26 mm) which was followed by PE (Cold) (8–18 mm), MeOH (10–15 mm), Hex (Hot) (10–12 mm) and CE extract (10–11 mm) at the dose of 2000 μg/well. At the dose of 1200 μg/well, EtOAc extract showed the highest activity against the Gram-positive bacteria (7–14 mm), PE (Cold) extract showed moderate (5–13) activity whereas, Hex (Hot), CE and MeOH extracts showed weak activity against all the Gram-positive bacteria. However, PE (Cold), Hex (Hot) and CE extracts did not produce any zone of inhibition against *Enterococcus faecalis*. Similarly, CE and MeOH extracts were also inactive against *Staphylococcus aureus.* Moreover, MeOH extract had no effect against *Bacillus subtilis.* Whereas, the standard drugs ciprofloxacin (5 μg/disc) and tetracycline (30 μg/disc) inhibited the growth of entire Gram-positive bacteria with a significant zone of inhibition 21–29 mm and 16–26 mm respectively.

**Table 5:** Summary of antimicrobial activity of *Anethum sowa* L. root extracts by diffusion method on Gram-positive bacteria

Extracts	Dose (μg/well)	Gram-positive bacteria and zone of inhibition in mm			
		*Bacillus subtilis*	*Bacillus cereus*	*Staphylococcus aureus*	*Enterococcus faecalis*
PE (Cold)	400	–	–	–	–
	1200	13.03±0.15	5.03±0.05	11.0±0.10	–
	2000	18.06±0.20	8.0±0.10	15.0±0.10	–
Hex (Hot)	400	–	–	–	–
	1200	7.03±0.15	–	7.1±0.10	–
	2000	10.0±0.20	–	12.0±0.10	–
CE	400	–	6.06±0.05	–	–
	1200	7.03±0.20	8.06±0.05	–	–
	2000	10.06±0.05	11.03±0.25	–	–
EtOAc	400	10.03±0.15	8.06±0.15	6.06±0.11	–
	1200	14.03±0.11	11.06±0.15	8.03±0.11	7.13±0.05
	2000	26.0±0.26	15.03±0.20	15.03±0.15	12.03±0.25
MeOH	400	–	–	–	–
	1200	–	7.06±0.11	–	8.0±0.10
	2000	–	15.06±0.15	–	10.03±0.25
Std. CP	5 μg/disc	27.0±0.10	29.0±0.20	25.10±0.10	21.13±0.11
Std. TE	30 μg/disc	24.0±0.10	21.03±0.15	26.03±0.11	16.03±0.15

*PE (Cold)* Petroleum ether cold extract, *Hex (Hot)* Hexane hot extract, *CE* Chloroform extract, *EtOAc* Ethyl acetate extract, *MeOH* Methanol extract, *Std. CP* Standard Ciprofloxacin, *Std. TE* Standard Tetracycline, “-” : no zone of inhibition. Each value is the mean± SD of three determinations (*n*=3)

**Table 6:** Summary of antimicrobial activity of *Anethum sowa* L. root extracts by diffusion method on Gram-negative bacteria

Sample	Dose (μg/well)	Gram-negative bacteria and zone of Inhibition in mm					
		*E.. coli 12079*	*E. coli 2799*	*P. aeruginosa*	*S. enteritidis* 1375	*S. typhi*	*A. aceti*
PE (Cold)	400	–	7.03±0.29	9.0±0.26	–	–	–
	1200	12.06±0.25	8.0±0.20	10.0±0.15	6.03±0.05	6.03±0.05	8.10±0.10
	2000	15.03±0.20	13.10±0.42	12.0±0.11	9.03±0.05	11.0±0.20	10.06±0.11
Hex (Hot)	400	–	–	–	–	–	–
	1200	–	6.0±0.10	8.0±0.17	–	–	7.03±0.15
	2000	–	9.03±0.12	11.0±0.11	–	–	11.03±0.25
CE	400	–	7.0±0.30	7.10±0.10	–	–	–
	1200	7.03±0.15	10.1±0.10	8.07±0.11	–	–	7.10±0.17
	2000	9.0±0.30	12.0±0.21	10.1±0.10	–	–	10.06±0.11
EtOAc	400	7.06±0.25	7.03±0.12	9.0±0.10	–	6.0±0.10	6.03±0.05
	1200	11.03±0.28	13.0±0.10	12.0±0.05	–	8.0±0.17	7.06±0.11
	2000	13.06±0.30	15.0±0.15	14.0±0.17	–	16.03±0.20	12.0±0.26
	400	–	–	–	–	–	–
	1200	6.03±0.05	7.03±0.06	9.03±0.05	–	–	9.06±0.20
	2000	8.06±0.20	10.0±0.17	11.0±0.10	–	7.06±0.05	12.16±0.15
Std. CP	5 μg/disc	25.03±0.40	23.10±0.17	29.0±0.11	39.0±0.17	35.03±0.37	28.03±0.23
Std. TE	30 μg/disc	9.03±0.30	11.0±0.21	16.10±0.15	23.0±0.10	25.03±0.20	23.06±0.28

*PE (Cold)* Petroleum ether cold extract, *Hex (Hot)* Hexane hot extract, CE: Chloroform extract, *EtOAc* Ethyl acetate extract, *MeOH* Methanol extract, *Std. CP* Standard Ciprofloxacin, *Std. TE* Standard Tetracycline, “-” :no zone of inhibition. Each value is the mean± SD of three determinations (*n*=3)

**Table 7:** Minimum inhibitory concentration (MIC) of *Anethum sowa* L. root extracts

Test organisms		MIC value (μg/mL)					
		PE (Cold)	Hex (Hot)	CE	EtOAc	MeOH	CP
Gram positive bacteria	*Bacillus subtilis*	125	62.5	125	62.5	250	1.55
	*Bacillus subtilis*	250	62.5	250	125	125	3.125
	*Staphylococcus aureus*	250	250	500	125	250	6.25
	*Enterococcus faecalis*	250	250	62.5	125	250	0.37
Gram negative bacteria	*Escherichia coli 12079*	500	500	500	62.5	250	0.75
	*Escherichia coli 2799*	250	250	62.5	62.5	250	0.37
	*Pseudomonas aeruginosa*,	1000	250	250	125	125	3.125
	*Salmonella enteritidis* 1375	125	250	250	62.5	125	0.37
	*Salmonella typhi*	250	250	250	125	125	0.19
	*Acetobacter aceti*	125	125	62.5	62.5	250	0.37

*PE (Cold)* Petroleum ether cold extract, *Hex (Hot)* Hexane hot extract, *CE* Chloroformextract, *EtOAc* Ethyl acetate extract, *MeOH* Methanol extract and *CP* Ciprofloxacin

**Table 8:** Minimum bactericidal concentration (MBC) of *Anethum sowa* L. root extracts

Test organisms		MBC value (μg/mL)					
		PE (Cold)	Hex (Hot)	CE	EtOAc	MeOH	CP
Gram-positive bacteria	*Bacillus subtilis*	250	125	250	125	500	12.5
	*Bacillus cereus*	500	125	500	500	500	25
	*Staphylococcus aureus*	500	500	1000	1000	500	50
	*Enterococcus faecalis*	1000	1000	125	500	500	100
Gram-negative bacteria	*Escherichia coli 12079*	1000	1000	1000	125	500	50
	*Escherichia coli 2799*	500	500	125	125	500	6.25
	*Pseudomonas aeruginosa*,	1000	500	500	250	250	25
	*Salmonella enteritidis* 1375	250	500	500	500	250	6.25
	*Salmonella typhi*	500	500	500	500	250	3.125
	*Acetobacter aceti*	500	250	125	250	500	50

*PE (Cold)* Petroleum ether cold extract, *Hex (Hot)* Hexane hot extract, *CE* Chloroform extract, *EtOAc* Ethyl acetate extract, *MeOH* Methanol extract and *CP* Ciprofloxacin

On the other hand, there was a significant variation was observed against the Gram-negative bacteria of the root extracts shown in Table 6. The EtOAc extract exhibited the highest activity (12–16 mm) followed by PE (Cold) (9–15 mm), MeOH (7–12 mm), CE (9–12 mm) and Hex (Hot) (9–11 mm) extracts at the dose of 2000 μg/well. Hex (Hot), CE, EtOAc and MeOH extracts had no susceptibility against *Salmonella enteritidis* 1375. Similarly, Hex (Hot) and CE extract showed the same against *Salmonella typhi* and also Hex (Hot) extract in *Escherichia coli 12079*. MeOH and Hex (Hot) extract showed registrant of all the Gram-negative bacteria at the dose of 400 μg/well. At the dose of 1200 μg/well, EtOAc (7–13 mm) and PE (Cold) (6–12 mm) extract showed moderate activity to the Gram-negative bacteria. Comparing the results with ciprofloxacin (5 μg/disc) and tetracycline (30 μg/disc), the entire Gram-negative bacteria inhibited to the extracts with a significant zone of inhibition 23–39 mm and 9–25 mm respectively. Again, the better activity was demonstrated with 2000 μg/mL concentration of all the tested extracts. The results demonstrate that Gram-positive bacteria are more sensitive to the extracts than the Gram-negative bacteria.

This higher resistance among Gram-negative bacteria could be due to the differences in the cell membrane of this bacterial group. The Gram-negative bacteria possess an outer membrane and a unique periplasmic space which is not found in the Gram-positive bacteria [46]. The resistance of Gram-negative bacteria towards antibacterial substances is related to the hydrophilic surface of their outer membrane which is rich in lipopolysaccharide molecules, presenting a barrier to the penetration of numerous antibiotic molecules and it is also associated with the enzymes in the periplasmic space, which are capable of breaking down the molecules introduced from outside [47]. The Gram-positive bacteria do not have such an outer membrane and cell wall structure. Antibacterial substances can easily destroy the bacterial cell wall and cytoplasmic membrane and result in a leakage of the cytoplasm and its coagulation [48]. However, MeOH extract did not show the trend completely in this study.

The MIC is the lowest concentration of the agent that completely inhibits visible growth, disregarding a single colony or a thin haze within the area of the inoculated spot [49]. *Anethum sowa* L. root extracts were tested with agar-dilution assay and susceptibilities were compared with a ciprofloxacin (positive control) at the different concentration by serial dilution technique. The results are shown in Table 7. The lowest MICs (62.5 μg/mL) was observed in EtOAc extract against *B. subtilis, E. coli 12079, E. coli 2799, S. enteritidis* 1375 and *A. aceti.* Moreover, the best activity possessed antibacterial activities with MICs of 62.5-125 μg/mL of EtOAc, CE and Hex(Hot) extracts followed by MeOH extract (125–250 μg/mL). However, PE (Cold), Hex (Hot) and CE extracts showed weak inhibition (MIC 500 μg/mL) in the test tubes containing *Escherichia coli 12079.* On the other hand, PE (Cold) extract showed the very weak against *Pseudomonas aeruginosa.* Overall, the Gram-positive bacteria showed the stronger activity than Gram-negative bacteria of the MIC experiment. However, standard ciprofloxacin showed the complete inhibition (i.e. no turbidity) against all of the test bacteria. *S. aureus* and *B. cereus* are well-known for being resistant to numerous antibiotics. Moreover, these organisms are capable of producing several types of enterotoxins that can cause septicaemia and several forms of enteritis. These findings are of promising in the case of EtOAc extract that was found to be active against these bacterial species.

In the MBC experiment (Table 8), the most susceptible bacteria to the EtOAc extract were *Bacillus subtilis, Escherichia coli 12079* and *Escherichia coli* 2799 followed by CE extract against *Enterococcus faecalis, Escherichia coli 2799* and *Acetobacter aceti* and Hex (Hot) extract against *Bacillus subtilis and Bacillus cereus* presenting an important growth of inhibition at the lowest concentration of MBC at 125 μg/mL. MeOH extract showed moderate effect against *Pseudomonas aeruginosa*, *Pseudomonas aeruginosa* and *Salmonella typhi* with MBC value of 250 μg/mL. Furthermore, PE (Cold), Hex (Hot), CE and EtOAc extract showed less potent activity at the dose of 1000 μg/mL. against *Staphylococcus aureus, Enterococcus faecalis, Escherichia coli 12079* and *Pseudomonas aeruginosa*. On the other hand, standard ciprofloxacin showed the most susceptible to Gram-positive and Gram-negative bacteria than the experimental extracts.

Microbial contamination still poses an important public health and economic concerns for human society. For these reasons, there has been increasing interest in searching of natural antimicrobial agents and it has notably increased for healthy lifestyles and healthy ageing. The screening of the chemical groups found in root extracts showed the presence of tannins, flavonoids and anthraquinone. The presence of these constituents at the high concentration may possess antimicrobial properties in the different extracts. Again, the antimicrobial effects of tannins take place by involving different mechanisms such as inhibition of extracellular microbial enzymes, deprivation of the substrates required for microbial growth or direct action on microbial metabolism through inhibition of oxidative phosphorylation [50]. Flavonoids and tannins are effective antimicrobial substances possibly due to their capability to form a complex with the extracellular and the soluble protein and to intricate with the cell membrane leading to the death of the bacteria [51]. Moreover, the plant extract was also positive for steroids which are very important compounds because it has a relationship with compounds such as sex hormone and also reported as antibacterial properties [52, 53]. The presence of the steroids in PE (Cold) extract justifies the antibacterial property of this plant part. Accordingly, the presence of these molecules in the active fractions might play an important role in the observed antibacterial activity. Their mode of action probably depends on the individual microorganism which could explain the large differences in MIC values between bacteria.

The antifungal activity of *Anethum sowa* root extracts were measured and represented in Table 9. The extracts did not show any growth of inhibition against *Aspergillus niger and Tricoderma sp.* except *Candida albincas* which showed some weak activity to the CE and MeOH extract. The results were compared with the standard drug, Fluconazole (100 μg/disc). The root extracts contain a number of secondary metabolites in the current studies. The isolation and purification of the extract may discover many significant novel antimicrobial lead compounds.

**Table 9:** Summary of antifungal activity of *Anethum sowa* L. root extracts by diffusion method

Test Fungi	PE (Cold)			Hex. Hot			CE			EtOAc			MeOH			Fluconazole		
	Zone (mm)	MIC	MBC	Zone (mm)	MIC	MBC	Zone (mm)	MIC	MBC	Zone (mm)	MIC	MBC	Zone (mm)	MIC	MBC	Zone (mm)	MIC	MBC
*Aspergillus niger*	–	–	–	–	–	–				–	–	–	–	–	–	–	–	–
*Candida albincas*		–	–	–	–	–	7	250	500		–	–		250	500		–	–
*Tricoderma sp.*	–	–	–	–	–	–				–	–	–	–	–	–	–	–	–

## Conclusion

Despite ongoing scientific research on this species, this study constitutes the first attempt to compile the phytochemical compositions as well as the antioxidant, antimicrobial and cytotoxic activities of *Anethum sowa* L. root extracts that could be found despite the throughout literature survey so far as we know. The knowledge of phytochemical constituents of the plant is the basic approach to identify novel secondary metabolites as un-modified form, semi-synthetic or drug templates. However, the mixture of biologically active compounds present in the different extract with different structures and also their synergistic effects may enhance the overall effect of the particular extract though their exact mode of action is poorly understood. This study delineates that methanol, ethyl acetate and chloroform extract could be a potentials in free-radical scavenging activity and cytotoxic effect and moderate antimicrobial activity. Since, crude methanol and ethyl acetate extract found to have moderate antibacterial activity. So, it can be assumed that different active secondary metabolites were present in these extracts. Furthermore, the activity of this plant constituent can help to elucidate the justification for the ethnomedicinal use of this plant species scientifically. Based on our findings, further studies are necessary to elucidate the mechanism lying with these effects of the plant extracts and could be open a new window in the search for new bioactive drug lead components of this plant extracts.

## Abbreviations

ASA: Ascorbic acid
BHT: Butylated hydroxytoluene
CE: Chloroform
DMSO: Dimethyl sulfoxide
DPPH: 2,2-diphenyl-1-picrylhydrazyl
EtOAc: Ethyl acetate
Hex: Hexane
MBC: Minimum bactericidal concentrations
MeOH: Methanol
MIC: Minimum inhibitory concentration
PE: Pet-ether
ROS: Reactive oxygen species
TSA: Tryptone soy agar
VcS: Vincristine sulfate
